# Accuracy of the Emergency Department Triage System using the Emergency Severity Index for Predicting Patient Outcome; A Single Center Experience

**DOI:** 10.30476/BEAT.2020.46452

**Published:** 2020-04

**Authors:** Raheleh Ganjali, Reza Golmakani, Mohsen Ebrahimi, Saeid Eslami, Ehsan Bolvardi

**Affiliations:** 1 *Medical Informatics, Department of medical informatics, Faculty of Medicine, Mashhad University of Medical Sciences, Mashhad, Iran*; 2 *Department of Emergency Medicine, Doctor Shariati Hospital, Mashhad University of Medical Sciences, Mashhad, Iran*; 3 *Department of Emergency Medicine, Mashhad University of Medical Sciences, Mashhad, Iran*; 4 *Pharmaceutical Research Centre, School of Pharmacy, Mashhad University of Medical Sciences, Mashhad, Iran *; 5 *Department of Medical Informatics University of Amsterdam, Amsterdam, The Netherlands*

**Keywords:** Triage, Emergency Severity Index (ESI), Patient outcome

## Abstract

**Objective:**

To evaluate the accuracy of the five-level triage system using the emergency severity index (ESI) and to determine the compliance of the triage level with patient outcomes.

**Methods:**

This was a cross-sectional study which was performed in the emergency department of Imam Reza Hospital of Mashhad during 2017. We included all the adult patients (≥15 years of age) referring to the emergency department. The data were recorded in a questionnaire containing three sections including demographic information, results of triage by ESI and final outcome of the patient. Patients referred to the triage unit were simultaneously triaged by triage nurse and some emergency medicine physicians. The triage was performed by a nurse with an emergency medicine physician (EMP) was considered as a gold standard and the outcome was compared in 24 hours later.

**Results:**

Overall, we included 400 patients with a mean age of 46.40 ± 18.52 years among whom there were 211 (52.8%) men and 189 (47.3%) women. Finally, 123 patients were hospitalized, 12 died, 256 were discharged by a physician, and 9 people left the hospital with their own consent. The calculated weight kappa was used to determine the agreement between the observers (nurse triage and physician) at 0.701 so that the agreement between the triage performed by a nurse and an EMP was in an excellent level (*p*<0.001). There was a significant relationship between the triage levels (determined by physicians) and the outcome of the patient (*p*<0.001), and the five-level system had a high overlap and significant relation with patient's outcome.

**Conclusion:**

The results of the current study revealed that the five-level triage system using the ESI has a high accuracy in triage and estimates the patient outcomes effectively and thus, could be used as an effective system in hospital triage.

## Introduction

The triage system is designed to categorize and classify the patients based on the severity of the signs and symptoms and also save the time for the time-sensitive conditions [1, 2]. The effectiveness triage is required when demand for medical care outstrips capacity, as has become commonplace in the emergency department (ED) due to overcrowding, now recognized as a major threat to patient safety and quality care across the globe [3-6]. Currently, the triage systems are being used in almost all the centers worldwide and their efficacy has been proved. However, the type of triage system used in each center is a matter of controversy [7, 8]. 

The most widely used ED triage tools employ a five-level triage scale and include the Australian Triage Scale, Canadian Emergency Department Triage and Acuity Scale (CTAS) [9], Manchester Triage Scale (MTS), and the Emergency Severity Index (ESI) [10]. ESI was developed in the USA and is being adopted by an increasing number of EDs globally [2, 4, 6, 10]. Despite its widespread adoption and numerous strengths that include ease of use and linkage to anticipated ED resource utilization [11], ESI has several limitations. It relies heavily on provider judgment and intuition, allowing for significant practice variation, with inter-rater reliability reported to range from k = 0.46 to 0.91 [12]. More than half of all visits in the USA are triaged to ESI level 3, generating a large pool of undifferentiated patients that creates challenges for efficient ED resource distribution and effective patient queuing, undermining the very purpose of triage [13]. Furthermore, ESI has never been well-validated against critical outcomes indicating time-sensitive needs in any setting [10].

The aim of this study was to evaluate the accuracy of the English version of the ESI and determine the degree of compliance of the triage level determined by the nurse and emergency medicine physicians as well as their degree of compliance with patient's outcome.

## Material and Method

*Study population*

This was a cross-sectional study being conducted during a 12-month period in 2017 Emergency Department of Imam Reza Hospital, a tertiary healthcare center affiliated with Mashhad University of Medical Sciences, Mashhad, Iran. We have included all the adult patients (≥15 years of age) who referred to the emergency department of our center during the study period. We included both patients were transferred to our center or those who primarily referred. Exclusion criteria were those who passed away less than 24 hours of admission and those with were dead on arrival. The study protocol was approved by the institutional review board (IRB) and medical ethics committee of the Mashhad University of Medical Sciences. All the included patients provided their informed written consents before inclusion in the study. 

*Triage level designations and gold standard comparator*

As a part of routine clinical care, ESI triage levels were assigned by a nurse with formal training in ESI for all patients at the time of ED arrival. Nurse-assigned ESI triage level was used to guide clinical care. For administrative purposes unrelated to this study, a second ESI triage level was entered for all patients at the close of ED encounter by the treating emergency physician. Both nurse and physician triage level designations were made according to the standardized ESI algorithm, but physician ESI level designation was made with full knowledge of actual ED resource utilization and acute clinical outcomes. Physician-assigned ESI triage level (assigned a posteriori) was used as a surrogate gold standard for accurate triage and was validated as such by the measurement of association with hospital admission and composite critical outcome. Prior to analysis, ESI triage scores were designated as high acuity (ESI level 1 or 2), moderate acuity (ESI level 3), or low acuity (ESI level 4 or 5). Redistribution of triage scores from five to three tiers was performed prior to analysis to more effectively capture the clinical impact of triage decisions, as ESI triage levels 1 and 2 are considered time-sensitive and are roomed immediately, while ESI level 3 patients often wait hours to receive definitive care, and ESI levels 4 and 5 are cared for in a separate area of the ED with a fast track designation. 

*Study protocol*

Prior to the study, a triage retraining course was conducted based on the severity index for all triage nurses. A guide to how performing triage was exposed in the presence of triage nurses. The nurses who were included in the study were unaware of the goals of the study. The names and personal information of the patients, the EMS and the nurses participating in the study were confidential and were only available to the researcher. The questionnaire used for collecting information consisted of three parts. The first part contained demographic information including age, sex, marital status, place of residence, and reason for referral to the emergency department. The second part contains the results of the triage and the third part contains the final result of the triage. The questionnaire was designed by two emergency medicine physicians. Patients referred to the emergency department were triaged by trained triage nurse and an emergency medicine physician. In the case of ill patients who needed life-saving interventions, the triage nurse was allowed to complete the triage form after initiating life-saving interventions. All patients were followed up within 24 hours and the outcome was determined. Patients were referred to the emergency department, the cardiopulmonary resuscitation room, the injured clinic, the fast track unit. Questionnaires were completed by patient records and section’s information documents. The triage performed by the emergency medicine physician was considered as the gold standard. Then the triage level determined by nurse was compared with gold standard.

*Statistical analysis*

Chi-square and Fisher's exact test were used for comparison of the ratio of different levels of triage determined by the nurse and gold standard. Also, the agreement between different levels of triage determined by the nurse and physician in emergency medicine was specifically evaluated and validated by regression analysis. Kappa weight index (K) was used to assess inter-rater reliability between physician and nurse triage. The Kappa index was less than 0.2 as poor agreement, 0.21-0.4 as a relatively weak agreement, 0.41-0.6 as the moderate agreements, 0.61-0.8 as a good agreement, and above 0.8, was considered as an excellent agreement.

## Results

From 400 triage cases, 59 patients (14.8%) were triaged by male and 341 patients (85.2%) by female nurses. The average age of the patients who participated in the study was 46.4 ± 18.52 years. Among the patients there were 211 (52.8%) men and 189 (47.2%) women. Overall, 318 patients were referred to the emergency department individually, 63 were transferred to the EMS, and 13 from other hospitals and 6 patients were referred to the ED by a university attending physician. The majority of the patients, 354 (88.5%) were dispositional in the first 6 hours, 30 patients (7.5%) at intervals of 6 to 12 hours, 11 patients (2.8%) in 12 to 24 hours and only 5 (1.2%) patients more than 24 hours (Table 1). Regarding the outcome, 123 patients were hospitalized, 12 died, 256 were discharged by a physician, and 9 people left the hospital with their own consent. There was no significant difference in the level of triage in the five-level triage performed by the emergency physician and the nurse at levels 1, 3 and 4, but at the 2 and 5 levels, this difference was statistically significant (Table 2).

The results of the comparison of triage nurse and an emergency physician are presented in Table 3. The calculated weight kappa was used to determine the agreement between the observers between nurse triage and physician at 0.701. The agreement between the triage performed by a nurse and a physician was in a high level (P <0.001). In the assessment of the three work shifts in the morning, evening and night, in the morning shift, with 135 cases of kappa coefficient of 0.696, in the evening work shift with 100 patients, 0.717 and at night work shift with 165 patients, 0.685, which was found to be the most agreeable work shift in the evening there have been. Nurses divided into three groups based on work experience in cases of triage by nurses with a work experience of less than 5 years Kappa coefficient was 0.714, in cases with history of 6 to 10 years and 0.703 in cases with more than 10 years it was 0.656 so that in all cases P<0.001 was obtained. A significant relationship was found between the different levels of triage performed by the nurse and the patient's outcome (*p*<0.001) and the five-level system had a high overlap and a significant effect on the patients' outcome. As 12 patients died and 21 were hospitalized at level one, only one patient was discharged at this level. At the second level, 55 patients were hospitalized and 6 patients were discharged. At level three, 41 patients were hospitalized and 11 patients were discharged, at level 4 only 6 patients were hospitalized and 213 were discharged. At level 5 all 25 patients were dismissed (Table 4).

There was also a statistically significant relationship between the different levels of triage performed by the physician and the outcome of the patient (P<0.001) and the five-level system had a high overlap and significant effect on the outcome of the patients. At the first level 15 patients were hospitalized and 11 patients died and only one patient was discharged at this level. At the second level, 52 patients were hospitalized and 1 patient died and 1 patient was discharged. At level three, 56 patients were hospitalized and 15 patients were discharged, at level 4 all 183 patients and at level 5 all 56 patients were discharged (Table 5).

## Discussion

ESI provides an innovative approach to the emergency department's triage by providing predictions about the number of facilities needed to deploy patients. The ESI predicts the differentiation of triage systems based solely on the severity of the disease. Of course, the allocation of facilities does not play a role for ill patients, such as the levels of 1 and 2 in ESI.

In our study, trained nurses in the emergency department for triage of patients were used. The calculated weight kappa was used to determine the agreement between the observers between nurse and physician triages at 0.701. The agreement between the triage performed by a nurse and an expert physician was in a high level.

In several studies, the reliability of the emergency severity index has been evaluated to measure the agreement between the observers. In a study at the Boston Hospital in 1998, researchers reported the validity and reliability of the emergency severity index. In this study, the nurse-appointed triage classes were strongly associated with the resources used in the emergency department and the outcomes, such as the rate of hospitalization. The reliability, between the researcher and the triage nurses was also good (77% of the same agreement and 22% with only one class difference) [14]. In the study of Tanabe *et al*. [15], agreement between the triage level which determined by the nurse and the correct level of triage was 0.89 and the correlation showed Pearson correlation 0.83 (*p*<0.001).On the other hand, in the study of Buschhorn *et al*. [16], the agreement between the triage level nurses and the emergency staff was moderate, and even after the training of pre-hospital emergency staff, the accuracy and precision of this method was still low. In the study of Platts-Mills *et al*. [17], the sensitivity and specificity of ESI triage in patients over 65 years of age requiring life-saving interventions were 42.3% and 99.2%, respectively.

The reason for the difference in sensitivity of ESI in Platts-Mills *et al*. [17] and our study is the difference in the method of doing research. In a study by Platts-Mills *et al*. [17], the validity has been measured by two nurses (an experienced nurse in the triage and a nurse in the emergency department). Also, the experienced nurse did not directly visited patients and only triaged by studying medical records. The results of Karimian *et al*. [18] showed that five-level triage has a high accuracy and precision in determining the therapeutic priorities of patients referred to the emergency department. The calculated kappa coefficient between nurse triage and physician was 0.87 (*p*<0.001).

Christ and colleagues evaluated the four triage systems, the Australian, Canadian, Manchester and ESI. At the end, concluded 5-level triage has priority in both validity and reliability to 3-level triage (*p*<0.001). A good and very good validity was reported for Canadian and ESI methods (0.7 to 0.95), while the Australian and Manchester methods were found to have an average score of 0.3 to 0.6 (6). In 2009, Storm-Versloot *et al*. [19] Also reviewed the compliance between the observers in the Manchester Triple System (MTS) and the Emergency Severity Index (ESI). This study reported that mismatches at one level for MTS and ESI were 10% and 22%, respectively, and mismatches for two levels were 1% and 2%, respectively. The weighting Kappa coefficients were 0.82 and 0.73, respectively [19].

In separation assessing of three shifts in the morning, evening and night, there was the most agreement in the evening Shift. In the study of Dehnadi Moghadam *et al*. [20], 30764 cases were the most frequent referring to the morning shift and the lowest referring to the evening shift. It seems that the lower amount of work in the evening shift can justify the higher agreement of the triage level in this work shift, and the high volume of referral in other shifts seems to reduce the accuracy of triage nurses. In examining the relationship between nursing triage and outcome of the patient, the five-level system had a high overlap and had a significant effect on the outcome of the patients. In the study of the relationship between the triage performed by the physician and the outcome of the patient, the five-level system had a high and significant overlap with the outcome of the patients. The results of Wuerz *et al*. [21] also confirmed our findings. The weighted Kappa was 0.8 (95% CI: 0.76-0.84) for allocate the triage. The facilities and the hospitalization were strongly correlated with the level of triage. For patients at level 5, only one quarter of patients (17 out of 67 patients) needed any testing or diagnostic process, and no illness was admitted. In contrast, at the level of one, only one in 12 patients was discharged, and none of them required less than 2 facilities.

In the study of Tanabe *et al*. [15], similar to our study, the level of triage with the outcome of the patient was completely related; hospitalization based on ESI level, 80% for level 1, 73% for level 2, and 51% for level 3, 6 Percentage was for level 4 and 5% for level 5. A higher percentage of patients at level 1 and 2 (40 and 12%) were admitted to the intensive care unit, and 3 out of 4 patients who died had triage levels 1 and 2. Karimian *et al*. [18] also showed that the five-level triage showed a high overlap with the outcome of patients, especially at levels one, two and five.

Main limitation of this study is a according to referring patients disposition, agreement of triage level between the nurse and the physician was not measurable, due to the fact that the number of reported levels in these cases was not equal, for example, there was no case in patients with personal refer in level triage 1 by physician, and also in the triage of patients referring with EMS, there was no patient at level 5, and Kappa coefficient was not measurable.

In conclusion, the results of the current study revealed that the five-level triage system using the ESI has a high accuracy in triage and estimates the patient outcomes effectively and thus, could be used as an effective system in hospital triage.

**Table. 1:** Baseline characteristics of included patients along with emergency department triage characteristics

**Variable **	**Value **
**Age (years)**	46.40 ± 18.52
**Gender **	
**Men (%)**	211 (52.8%)
**Women (%) **	189 (47.3%)
**Working Shifts **	
**Morning (%)**	135 (33.8%)
**Afternoon (%)**	100 (25.0%)
**Night (%)**	165 (41.3%)
**Referral type **	
**Personnel (%)**	316 (79.0%)
**EMS (%)**	65 (16.3%)
**Other hospitals (%)**	13 (3.3%)
**Attending physician refer (%)**	6 (1.5%)
**Triage nurse gender **	
**Men (%)**	59 (14.8%)
**Women (%)**	341 (85.3%)
**Triage nurse age **	
**<30 years (%)**	62 (15.5%)
**30-40 years (%)**	337 (84.3%)
**40-50 years (%)**	1 (0.2%)
**ED Deposition **	
**<6 hours (%)**	354 (88.5%)
**6-12 hours (%) **	30 (7.5%)
**12-24 hours (%)**	11 (2.8%)
**>24 hours (%)**	5 (1.3%)
**Outcome **	
**Admission (%)**	124 (31.0%)
**Death (%)**	12 (3.0%)
**Discharge (%)**	256 (64.0%)
**Consent discharge (%)**	8 (2.0%)

**Table. 2:** Distribution of five-level triage nurses and physicians in patients referred to our emergency department

**Triage level**	**Nurse **	**Emergency Physician**	**P value **
One	34 (8.5%)	27 (6.8%)	0.771	
Two	65 (16.2%)	55 (13.8%)	**0.041**	
Three	57 (14.2%)	79 (19.8%)	0.153	
Four	219 (54.8%)	183 (45.8%)	0.186	
Five	25 (6.2%)	56 (14.0%)	**0.002**	

**Table. 3:** Comparison of the level of five-level triage set by nurse with gold standard (emergency physician)

**Triage Level **		** Emergency Physician**				
		**Level 1**	**Level 2**	**Level 3**	**Level 4 **	**Level 5**
**Nurse **	**Level 1**	26	8	0	0	0
	**Level 2**	1	44	20	0	0
	**Level 3**	0	1	18	8	0
	**Level 4**	0	2	11	175	31
	**Level 5**	0	0	0	0	25

**Table. 4:** Overlapping of different levels of triage determined by the nurse with the outcome

**Triage Level**		**Outcome **			
		**Hospitalization**	**Death**	**Discharge**	**Consent Discharge**
**Nurse**	**Level 1**	21	12	1	0
	**Level 2**	55	0	6	4
	**Level 3**	41	0	11	5
	**Level 4**	6	0	213	0
	**Level 5**	0	0	25	0

**Table. 5:** Overlapping of different levels of triage determined by the emergency physician (EMP) with the outcome

**Triage Level**		**Outcome **			
		**Hospitalization**	**Death**	**Discharge**	**Consent Discharge**
**Emergency physician **	**Level 1**	15	11	1	0
	**Level 2**	52	1	1	1
	**Level 3**	56	0	15	8
	**Level 4**	0	0	183	0
	**Level 5**	0	0	56	0

## Conflict of interest:

None 

## References

[1] Bazm A, Khorasani E, Etemadi M, Nadeali H (2015). Improving Five-level Triage Form According to the Experts Viewpoint; A Qualitative Study. Bull Emerg Trauma.

[2] Ghafarypour-Jahrom M, Taghizadeh M, Heidari K, Derakhshanfar H (2018). Validity and Reliability of the Emergency Severity Index and Australasian Triage System in Pediatric Emergency Care of Mofid Children's Hospital in Iran. Bull Emerg Trauma.

[3] Daemi A (2016). The Role of Electronic Triage System in Management of Hospital Emergency Department. Bull Emerg Trauma.

[4] Hinson JS, Martinez DA, Schmitz PSK, Toerper M, Radu D, Scheulen J (2018). Accuracy of emergency department triage using the Emergency Severity Index and independent predictors of under-triage and over-triage in Brazil: a retrospective cohort analysis. Int J Emerg Med.

[5] Pourasghar F, Daemi A, Tabrizi JS, Ala A (2015). Inter-rater Reliability of Triages Performed by the Electronic Triage System. Bull Emerg Trauma.

[6] Pourasghar F, Tabrizi JS, Ala A, Daemi A (2016). Validity of the Electronic Triage System in Predicting Patient Outcomes in Tabriz, Iran: A Cross-Sectional Study. Bull Emerg Trauma.

[7] Trinder MW, Wellman SW, Nasim S, Weber DG (2018). Evaluation of the trauma triage accuracy in a Level 1 Australian trauma centre. Emerg Med Australas.

[8] Varshney K, Mallows J, Hamd M (2012). Disaster triage tags: is one better than another?. Emerg Med Australas.

[9] Stobbe K, Dewar D, Thornton C, Duchaine S, Tremblay PM, Howe D (2003). Canadian Emergency Department Triage and Acuity Scale (CTAS): Rural Implementation Statement. Cjem.

[10] Gilboy N, Tanabe T, Travers D, Rosenau AMJR (2011). MD: Agency for Healthcare Research, Quality. Emergency Severity Index (ESI): A triage tool for emergency department;.

[11] McHugh M, Tanabe P, McClelland M, Khare RK (2012). More patients are triaged using the Emergency Severity Index than any other triage acuity system in the United States. Acad Emerg Med.

[12] Christ M, Grossmann F, Winter D, Bingisser R, Platz E (2010). Modern triage in the emergency department. Dtsch Arztebl Int.

[13] Dugas AF, Kirsch TD, Toerper M, Korley F, Yenokyan G, France D (2016). An Electronic Emergency Triage System to Improve Patient Distribution by Critical Outcomes. JEmerg Med.

[14] Selker HP, Beshansky JR, Griffith JL, Aufderheide TP, Ballin DS, Bernard SA (1998). Use of the acute cardiac ischemia time-insensitive predictive instrument (ACI-TIPI) to assist with triage of patients with chest pain or other symptoms suggestive of acute cardiac ischemia A multicenter, controlled clinical trial. Ann Intern Med.

[15] T Tanabe P, Gimbel R, Yarnold PR, Kyriacou DN, Adams JG (2004). Reliability and validity of scores on The Emergency Severity Index version 3. Academic emergency medicine.

[16] Buschhorn HM, Strout TD, Sholl JM, Baumann MR (2013). Emergency medical services triage using the emergency severity index: is it reliable and valid?. J Emerg Nurs.

[17] Platts-Mills TF, Travers D, Biese K, McCall B, Kizer S, LaMantia M (2010). Accuracy of the Emergency Severity Index triage instrument for identifying elder emergency department patients receiving an immediate life-saving intervention. Acad Emerg Med.

[18] Kariman H, Joorabian J, Shahrami A, Alimohammadi H, Noori Z, Safari S (2013). Accuracy of emergency severity index of triage in Imam Hossein hospital-Tehran, Iran (2011). Journal of Gorgan University of Medical Sciences.

[19] Storm-Versloot MN, Ubbink DT, Chin a Choi V, Luitse JS (2009). Observer agreement of the Manchester Triage System and the Emergency Severity Index: a simulation study. Emerg Med J.

[20] Dehnadi MA, Yousefzadeh S, Hemati H, Shaabani S (2008). Comparison the number of triaged patients in three working shift in poursina hospital in rasht.

[21] Wuerz RC, Milne LW, Eitel DR, Travers D, Gilboy N (2000). Reliability and validity of a new five‐level triage instrument. Academic emergency medicine.

